# Can Large Language Models Generate Outpatient Clinic Letters at First Consultation That Incorporate Complication Profiles From UK and USA Aesthetic Plastic Surgery Associations?

**DOI:** 10.1093/asjof/ojad109

**Published:** 2023-12-06

**Authors:** Richard H R Roberts, Stephen R Ali, Thomas D Dobbs, Iain S Whitaker

## Abstract

The importance of written communication between clinicians and patients, especially in the wake of the Supreme Court case of Montgomery vs Lanarkshire, has led to a shift toward patient-centric care in the United Kingdom. This study investigates the use of large language models (LLMs) like ChatGPT and Google Bard in enhancing clinic letters with gold-standard complication profiles, aiming to improve patients’ understanding and save clinicians’ time in aesthetic plastic surgery. The aim of this study is to assess the effectiveness of LLMs in integrating complication profiles from authoritative sources into clinic letters, thus enhancing patient comprehension and clinician efficiency in aesthetic plastic surgery. Seven widely performed aesthetic procedures were chosen, and complication profiles were sourced from the British Association of Aesthetic Plastic Surgeons (BAAPS) and the American Society of Plastic Surgeons (ASPS). We evaluated the proficiency of the ChatGPT4, ChatGPT3.5, and Google Bard in generating clinic letters which incorporated complication profiles from online resources. These letters were assessed for readability using an online tool, targeting a recommended sixth-grade reading level. ChatGPT4 achieved the highest compliance in integrating complication profiles from BAAPS and ASPS websites, with average readability grades between eighth and ninth. ChatGPT3.5 and Google Bard showed lower compliance, particularly when accessing paywalled content like the ASPS Informed Consent Bundle. In conclusion, LLMs, particularly ChatGPT4, show promise in enhancing patient communications in aesthetic plastic surgery by effectively incorporating standard complication profiles into clinic letters. This aids in informed decision making and time saving for clinicians. However, the study underscores the need for improvements in data accessibility, search capabilities, and ethical considerations for optimal LLM integration into healthcare communications. Future enhancements should focus on better interpretation of inaccessible formats and a Human in the Loop approach to combine Artifical Intelligence capabilities with clinician expertise.

**Level of Evidence: 3:**

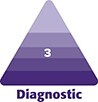

Clear and accurate written communication with patients remains paramount in plastic surgery practice. The landmark Supreme Court case of Montgomery vs Lanarkshire has profoundly impacted the consent process in the United Kingdom, advocating a paradigm shift from a paternalistic approach to a patient-centric model.^1^ This approach emphasizes the importance of fully informing patients about potential complications.^2^ However, clinicians often face time constraints when communicating these in the clinic environment and including them in letters to patients. Thus, there is a growing need for innovative solutions to facilitate the integration of gold-standard complication profiles into the consenting process.

Recently, novel applications of large language models (LLMs), including popular models such as ChatGPT (OpenAI, San Francisco, CA) and Google Bard (Google, Menlo Park, CA), have showcased their ability to understand and generate coherent human-like text.^3,4^ These models recognize language structure, classify content, and generate contextually appropriate responses. This facilitates a cooperative strategy, wherein the language model serves as an auxiliary instrument, offering drafts which can be subsequently adapted and tailored. This collaborative technique strives to augment productivity while preserving the human expert's intricate comprehension and individualized approach.

To advance the existing discussion surrounding the application of LLMs in cosmetic surgery which includes using ChatGPT to create clinic letters and identifying novel systematic review topics,^5,6^ we wish to assess LLM’s ability in generating clinic letters that incorporate meticulously curated complication profiles. The secondary aim was to assess LLM's ability to present this information comprehensibly and in a reader-friendly manner, enhancing patients’ understanding. These aims build further upon the recommendations of the Topol review,^7^ which Artifical Intelligence (AI) can begin to liberate clinicians from administrative tasks, thereby allocating more time for patient interaction.

## METHODS

**Figure 1.**
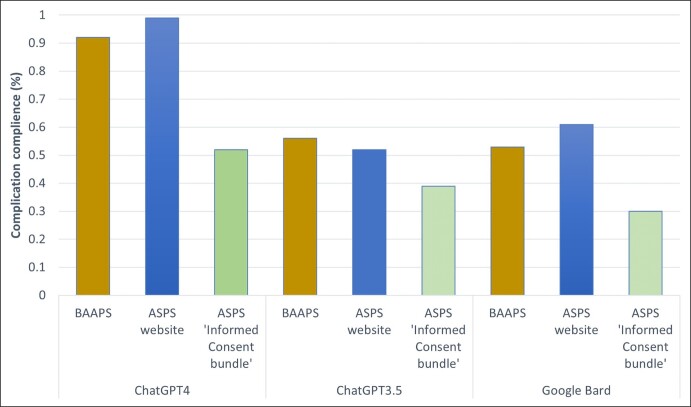
Comparing complication compliance between ChatGPT4, Chat GPT3.5, and Google Bard retrieving complication profiles from the 3 different sources.

**Figure 2.**
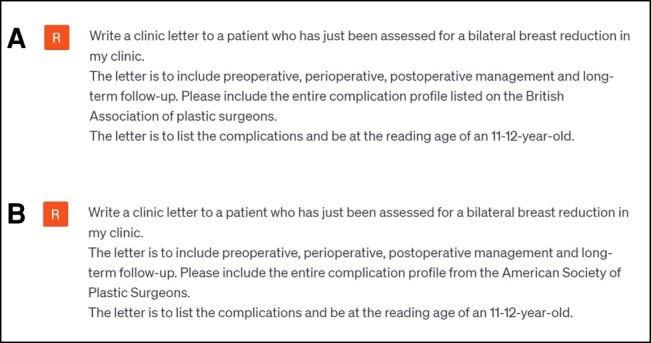
Example prompts used to create a clinic letter which included the risk and complication profile from (a) BAAPS and (b) ASPS used for ChatGPT3.5 and ChatGPT4.

The study selected 7 widely performed aesthetic procedures: bilateral breast augmentation, bilateral breast reduction (BBR), blepharoplasty, abdominoplasty, liposuction, facelift, and rhinoplasty. Complication profiles for these procedures were sourced from the freely accessible resources provided by the British Association of Aesthetic Plastic Surgeons^8^ (BAAPS) and the American Society of Plastic Surgeons^9^ (ASPS) on their respective websites. Additionally, ASPS offered a comprehensive list of complications behind a paywall in the form of a downloadable PDF titled “2020 Informed Consent Bundle”.^10^ To evaluate proficiency, 2 distinct prompts (Figure 1) were structured for each procedure—1 based on the BAAPS complication profile and the other referencing the ASPS resources. European guidelines dictate that numerical statistics should accompany verbal descriptors when communicating risk.^11^ However, evidence suggests that risks conveyed in numerical form without clinician interaction can cause patients to misinterpret statistics, applying higher emotional weight to numerical values and therefore impeding informed decision making.^12,13^ Considering this, numerical probabilities of complication requests were not included within the prompts. From March 3 to April 8, 2023, these prompts requested the LLMs to outline the preoperative, perioperative, and postoperative care while incorporating the relevant complication profile from the respective association (Figure 2). We evaluated the proficiency of the LLMs in incorporating the individual complications as encapsulated within the BAAPS and ASPS profiles. We quantified the complication compliance by determining the percentage of the complication profile that was incorporated within the generated letters.

In line with health-related patient communication standards within the United States of America, it is recommended that written material should be comprehensible to individuals at or below a sixth-grade reading level (age 11-12).^14^ To evaluate the readability of the generated clinic letters, we employed the widely accepted online tool, https://readable.com. This resource utilizes several readability formulas commonly used in various studies.^15,16^ Subsequently, mean scores were calculated for each LLM, enabling a comprehensive assessment of readability.

## RESULTS

Forty-two hypothetical clinical letters were created using the 3 separate models. The readability scores suggest that the letters may be suitable for varying reading abilities. The average reading age for ChatGPT3.5 was USA eighth grade (UK 13-14 years old) and ChatGPT4 and Bard was USA ninth grade (UK 14-15 years old) and considered by the United States Department of Health and Human Services (USDHHS) as “average difficulty.”

**Figure 3.**
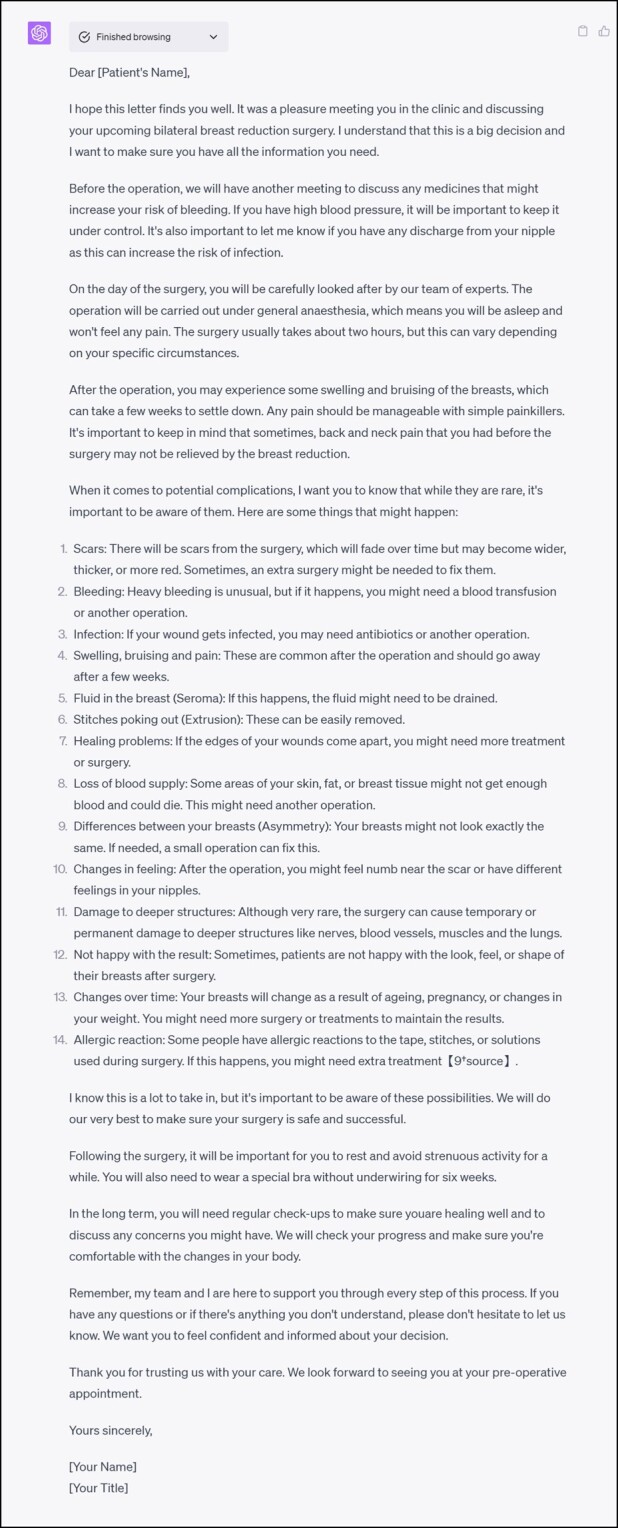
Example clinic letter created using ChatGPT4 incorporating the BAAPS complication profile.

Complication compliance varied between all 3 models (Figure 3). The ChatGPT4 model performed well, achieving an average BAAPS complication score of 0.92 CI 95% (0.78-1.00) and ASPS website average of 0.99 CI 95% (0.93-1.00). Nevertheless, when it came to delineating and conveying the complication profiles derived from the ASPS Informed Consent Bundle, the ChatGPT4 model exhibited a mean performance rating of 0.52 CI 95% (0.42-0.62).

The Chat GPT3.5 model showed different performance characteristics. It had an average complication compliance BAAPS score of 0.56 CI 95% (0.49-0.63), an ASPS website compliance of 0.52 CI 95% (0.45-0.59), and an ASPS gold-standard compliance of 0.39 CI 95% (0.26-0.53).

Finally, we analyzed Google Bard. This had a BAAPS average complication compliance of 0.53 CI 95% (0.47-0.59), an ASPS website compliance of 0.52 CI 95% (0.45-0.59), and an ASPS gold-standard compliance of 0.30 CI 95% (0.26-0.34).

## DISCUSSION

The results of our study reveal varying levels of accuracy and readability among the evaluated LLMs in generating clinic letters for aesthetic plastic surgery. All models generated letters of “average difficulty” in terms of their readability according to the guidelines set by the USDHHS, indicating that most patients could comprehend the content, but that it was more difficult than the ideal.

However, their adherence to gold-standard complication profiles differed significantly. Notably, our study found that the ChatGPT4 model exhibited a remarkable level of precision in interpreting and incorporating gold-standard complication profiles sourced from both the BAAPS and the ASPS websites. In contrast, the Google Bard model underperformed relative to expectations, with the LLM seemingly unable to search and implement targeted information. It is important to acknowledge that both ChatGPT and Google Bard encountered significant challenges in incorporating exact complication profiles without effective real-time web access which was offered by ChatGPT4. Despite the ChatGPT LLMs being trained on the 2021 “common crawl” dataset,^17^ which included both respective websites, the nature of text generation of LLMs means that they were unable to extract and implement this exact information. The web access integration of ChatGPT4 negated these issues by searching and implementing in real-time based on the prompts provided.

We observed an inability of all 3 LLMs to access the ASPS Informed Consent Bundle protected by a paywall, therefore, failing to be trained on this pertinent data. Similar challenges were faced by the web access integration of ChatGPT4 and Google Bard in bypassing the paywall or processing the PDF format. A potential future direction for LLMs is to refine their capacity to search, access, and interpret PDF content without the use of third-party applications. Ensuring that LLMs have immediate access to high-quality information is vital for effective integration into the healthcare sector. Consequently, we speculate that paywalls could hinder future LLM applications, requiring mitigation strategies. This insight promotes the open sourcing of such clinical data as a step toward broadening the application and utility of LLMs in the clinical environment.

It is important to note that there is ongoing concern that LLMs such as the ChatGPT models and Google Bard still make mistakes in terms of clinical knowledge,^18^ are unable to incorporate image data,^19,20^ and have ethical and privacy concerns.^21^ An example of a clinical error is illustrated in the letter provided (Figure 3). ChatGPT labeled all complications as “rare” which included wound-site infections. Wound-site infections, which can range from wound exudate to severe wound-site infections and sepsis, are observed in 16% of BBRs.^22^ Under European guidelines, this would be defined as “very common.”^11^ However, while this represents a response error, the quality of the prompt has been highlighted as a major determinant in response accuracy^23^ and could be a contributing factor.

To enhance the uniformity of correspondence in future works regarding clinical letters, it is crucial to direct further attention toward the refinement of LLMs. We propose a Human in the Loop (HITL) approach, where the language model serves as an initiating platform, furnishing a basic draft that the surgeon can then refine and individualize. AI systems can learn from human inputs and corrections over time, adapting and improving its performance.^24^ This collaborative mechanism is aimed at elevating efficiency while preserving the surgeon’s nuanced comprehension and personal touch in communication.

Our study underscores the potential of LLMs in facilitating patient–physician communication through the generation of clear, readable, and accurate clinic letters which aligns with the patient-centered approach mandated by the Montgomery case. We propose further exploration of LLM applications in healthcare, particularly in light of the increasing emphasis on patient-centered care and informed consent. However, it is crucial to consider the complexities of medical language and the significance of contextual understanding in achieving effective communication.

## CONCLUSION

This study highlights the transformative potential of LLMs in enhancing patient communication within aesthetic plastic surgery. By integrating BAAPS and ASPS complication profiles, LLM-generated clinic letters provide accurate and relevant information, saving time for clinicians. This approach empowers patients to make informed decisions while ensuring compliance with legal standards while providing time-saving benefits for clinicians which contribute toward improved workflow management.
